# Functional Traits Plasticity of the Invasive Herb *Argemone ochroleuca* Sweet in Different Arid Habitats

**DOI:** 10.3390/plants9101268

**Published:** 2020-09-26

**Authors:** Abdulaziz M. Assaeed, Saud L. Al-Rowaily, Magdy I. El-Bana, Ahmad K. Hegazy, Basharat A. Dar, Ahmed M. Abd-ElGawad

**Affiliations:** 1 Plant Production Department, College of Food & Agriculture Sciences, King Saud University, P.O. Box 2460, Riyadh 11451, Saudi Arabia; assaeed@ksu.edu.sa (A.M.A.); srowaily@ksu.edu.sa (S.L.A.-R.); baseratali@gmail.com (B.A.D.); 2 Botany Department, Faculty of Science, Port Said University, Port Said 42511, Egypt; magdyelbana@sci.psu.edu.eg; 3 Department of Botany and Microbiology, Faculty of Science, University of Cairo, Giza 12613, Egypt; hegazy@sci.cu.edu.eg; 4 Department of Botany, Faculty of Science, Mansoura University, Mansoura 35516, Egypt

**Keywords:** mexican poppy, noxious weeds, conservation, resource allocation, arid lands, disturbed habitat

## Abstract

Understanding the strategies and mechanisms of invasive species could guide their control and management especially in arid ecosystems. This study compares the vegetative and reproductive functional traits of the invasive Mexican poppy (*Argemone ochroleuca*), in seven habitat types, in southwestern Saudi Arabia. The results showed that the aboveground phenological attributes such as plant height, leaf area, and leaf dry mass attained the highest values in the wadi channels, whereas these attributes attained the lowest values in the mountain ranges. Maximum specific leaf area, root parameters, and all reproductive traits were recorded in the abandoned fields. In contrast to all other habitats, populations from abandoned fields had a greater investment of resources in belowground structures, while the population growing in the wadi channels and mountain ranges habitat allocated more energy to vegetative parts. The plasticity in vegetative and reproductive resource allocation in *A. ochroleuca* is an important mechanism in determining its colonizing ability in different habitat types and expanding the distribution range. The present data of the functional traits of *A. ochroleuca* agree with the resource fluctuation hypothesis, where the plant flourished in the abandoned fields that attained the highest values of organic matter and nutrients. Therefore, the restoration of these disturbed habitats could improve the resistance toward invasion by this noxious weed.

## 1. Introduction

Invasive alien plants have become a serious threat to native plant diversity and ecosystem function in many regions of the world [1]. A major challenge for plant ecologists is to highlight how invasive species respond to the variation in environmental conditions [2,3]. Successful invaders often demonstrate high levels of plasticity that allow them to succeed in a wide range of habitats [4,5,6,7]. Thus, comparing the relative performance of potential invaders in different habitats may help in understanding their strategies and mechanisms that could guide their control and management, especially in the fragile ecosystems [8].

Previous studies have demonstrated that functional traits of invasive species often vary across different habitat types [9,10,11,12]. Variations of plant shoot and root system characteristics are considered good morphological attributes for predicting successful invasion among different habitat types [13,14,15]. In addition, several studies indicated that in some habitats, invasive species may show higher values of specific leaf area (SLA) and leaf dry matter content (LDMC) than in other habitats [14,16]. These foliar attributes are considered effective functional traits as they are expected to be predictors of both plant strategy and fast resource capture and maybe the primary process influencing successful invasions [17,18]. For example, greater SLA can increase the ability to capture and utilize resources that can lead to an increased growth rate and overall fitness [6].

Alternatively, reproductive traits of invasive species change due to variations in ecological conditions and biophysical constraints within different habitats [5,18]. Invasive species may be found at higher densities and with higher reproductive rates in some habitats than others [19]. Habitats with increased resources tend to be more productive for invasive species with higher reproductive output and higher rates of spread [20]. In addition, invasive species cope with habitat changes by increasing biomass allocation to reproductive structures [5,21]. Plants adjust allocation of carbon assimilates between their organs to ensure the persistence of the species in a specific habitat [22]. Invasive species are usually physiologically plastic, allowing them to adapt to a variety of habitats and assure successful invasion [18,23,24].

Arid environments are considered as the ecosystems least affected by biological invasions [25]. However, in recent years, many invasive species have predominantly invaded managed and/or disturbed areas, such as agricultural fields and roadsides [2,26]. Furthermore, some of these species have escaped to many natural habitats including wetlands, rangelands, and biodiversity hotspots, and can generate significant negative impacts on ecosystem processes and functions [2,27,28]. Given the wide expansion of many invasive species across a range of desert habitats, more studies are required on their morphological and reproductive attributes that promote their spreading and invasion success.

The Mexican poppy, *Argemone ochroleuca* Sweet (Papaveraceae), is a worldwide invasive annual herb native to Central America. It grows in a wide range of soil types but normally dominates sandy soils, and can grow well in nutrient-poor soils. It is naturalized in several semiarid regions. The seeds are dispersed through wind, water, birds, and agricultural practices. In Mexico, *A. ochroleuca* is used in traditional medicine to treat several diseases [29]. Today, this weed invades many countries of Africa, Asia, Europe, Oceania, North America, and South America [30,31,32]. In the southwest of Saudi Arabia, the species has invaded a wide range of habitat types including agro-ecosystems, fallow lands, roadsides, wastelands, wetlands, gravelly and sandy plains [32,33,34]. The spread of *A. ochroleuca* has been ascribed to disturbance, overgrazing, and invasive competency [32,34]. The *Mexican poppy* has been reported to possess allelopathic activity which enables it to invade and dominate new habitats [33,35].

This study explores how different vegetative (shoot height, canopy diameter, ramifications, root length, root dry mass, leaf area, leaf dry mass, SLA, leaf thickness, seeds/fruit, root mass fraction, vegetative mass fraction, and reproductive mass fraction) and reproductive traits (flowers/individual, fruits/individual, and fruit dry mass), correlate to the successful invasion of *A. ochroleuca* in different habitats. We expect that plant functional traits vary with different habitat types as an intraspecific divergence and plasticity to different environmental conditions. The objectives of this study are to: (1) compare the vegetative traits related to resource capture, growth rate and competitive ability across different habitats; (2) determine biomass allocation to roots, vegetative and reproductive plant parts; (3) identify the possible edaphic factors that may influence the relative resource allocation performance of *A. ochroleuca* across different habitat types. 

## 2. Materials and Methods

### 2.1. The Study Area

**Figure 1:**
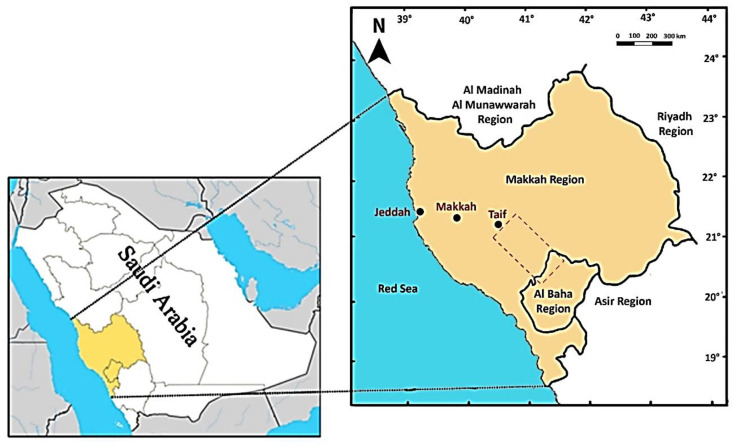
Location of the study area, the dashed polygon extends south from Taif in Makkah province to the northern edge of Baha province, Saudi Arabia.

The study was carried out in an area running from Al-Taif to Al-Baha in the southwest of Saudi Arabia with a distance of 245-km-long (Figure 1). The area is largely dominated by rugged escarpment and drainage streams (wadis). However, many sites have sandy and alluvial plains where agriculture is the main activity. The area lies within the tropical and arid climate with an average annual rainfall of 165 mm [36]. Rainfall is most predictable in the spring and summer but can occur during any month of the year when moist air is forced up the escarpment from the Red Sea [37]. Most of the rainfall is associated with intense thunderstorms [38]. Such heavy rain storms lead to the formation of many temporary ponds that can exist for more than one month. The average air temperature is 16.5 °C in winter and 29.1 °C in summer. 

### 2.2. Habitat Types and Soil Analysis

Populations of *A. ochroleuca* were surveyed in seven habitats: abandoned fields, roadsides, cultivated fields, wadi channels, wetlands, mountain ranges, and sandy plains. For each habitat, we randomly selected ten plots (5 m × 5 m) at the peak of the growing season (spring) for later measurements. Soil samples were collected randomly from three different locations (0 to 30 cm depth) within each plot and subsequently pooled for each plot. Soil samples were air-dried at room temperature before oven drying at 70 °C and sieved through a 2-mm sieve. Soil texture was determined by the hydrometer method, providing quantitative data on the percentage of sand, silt, and clay [39]. Soil organic matter (OM) was determined by wet combustion with dichromate at 450 °C [40]. Soil water extracts (1:5) were prepared for the estimation of soil electrical conductivity (EC) and pH [40]. Soluble inions (Cl and SO_4_) were determined by titration method, while soluble cations (Ca, Mg, Na, and K) were determined using a flame photometer according to Rhoades [41]. Available phosphorus was determined colorimetrically as described by Nelson and Sommers [42]. Available nitrogen was determined by the Kjeldahl method as described by Bremner and Mulvaney [43].

### 2.3. Morphological Traits Measurements

For each selected plot in each habitat, at the end of the flowering–fruiting stage, we recorded the morphological traits of 15 individuals of *A. ochroleuca* related to shoot density for shoot height from the ground, shoot canopy diameter (based on 2–3 measurements/individual), and the number of secondary and tertiary ramifications, i.e., 1050 individuals were screened (15 individuals per plot × 10 plots per habitats × 7 habitats). After these measurements, 15 mature and healthy individuals were excavated and brought to the laboratory in an ice-cool box. Roots were manually washed and their length and dry mass were measured for each individual. Leaf area (one-sided projected area) was measured on the fresh longest full-grown leaves of each individual using a portable laser leaf area meter CI-202 scanning planimeter (CID Inc, Camas, WA, USA). Plants were separated into roots, stems, and leaves and dried at 70 °C for 48 h. Based on these measurements, the specific leaf area (SLA) was determined as the ratio of leaf area to leaf dry mass. Leaf dry matter content (LDMC) was determined as the ratio of leaf dry mass to saturated fresh mass. Leaf thickness was estimated from the ratio SLA × LDMC^−1^ as described by Vile, et al. [44]. These functional traits were selected according to Pérez-Harguindeguy, et al. [45], to assess the response and plasticity of *A. ochroleuca* to the environmental factors within different habitats

### 2.4. Reproductive Traits Measurements

In each measured plot within each habitat, reproductive traits were assessed for the selected 15 mature individuals. In the field, we counted the number of flowers and fruits per individual. After counting, the fruits were separated from the shoot and placed in paper bags. In the laboratory, we used a subsample to count the number of seeds per fruit. The fruit dry mass was measured.

### 2.5. Statistical Analysis

One-way analyses of variance (ANOVAs) were used to examine the significant difference among the different habitats (*n = 7*) regarding the soil properties as well as the functional traits of *A. ochroleuca*. SPSS software (Version 24.0; SPSS, Inc., Chicago, IL, USA) was used to perform statistical analysis. Two data matrices of different functional traits, as well as the soil variables within the studied habitats, were prepared. According to the approach proposed by Legendre and Legendre [46], the data of the vegetative and reproductive traits from the seven habitats were subjected to principal component analysis (PCA), to generate a synthetic representation of the habitats and the functional traits. The functional traits were plotted as loading vectors in the bi-plot, while the habitats were plotted as observations. The relationships between the soil variables, habitats, and functional traits were assessed by ordination analysis using canonical correspondence analysis (CCA). Additionally, the agglomerative hierarchical clustering (AHC) and heatmap were performed based on the data of soil variables as well as the functional traits of *A. ochroleuca* populations within the seven studied habitats. The AHC was performed based on the Pearson correlation coefficient and weighted pair-group average agglomeration method. PCA and AHC were performed using XLSTAT software program (version 2018, Addinsoft, NY, USA), while CCA was produced using the MVSP software program, ver. 3.1 [47].

## 3. Results

### 3.1. Morphological Traits Variations among the Habitats of A. ochroleuca

All studied vegetative and reproductive traits of *A. ochroleuca* were significantly different among habitats (Table 1). The habitat of wadi channels had the highest values of vegetative height, canopy diameter, number of secondary ramifications, leaf area, leaf dry mass, while the lowest values of these parameters were recorded in populations of the mountain ranges. 

For example, the height, ramifications, and canopy diameter of the wadi channels habitat were 3.1-, 2.7-, and 2.1-fold higher than those recorded in the mountain ranges habitat, respectively (Table 1). The highest values of roots data (root length and dry mass) were recorded in abandoned fields habitat, while the lowest values were recorded in the populations of the mountain ranges habitat. For example, the root dry mass of *A. ochroleuca* populations in the abandoned fields habitat was about 3-fold of that in mountain ranges habitat. The wadi channels habitat attained the highest values of leaf area (116.77 cm^2^) and leaf dry mass (2077.70 mg), while the highest value of leaf thickness (179.07 µm) was recorded for the *A. ochroleuca* populations growing in sandy plains habitat. The specific leaf area was more variable among habitats, with the highest and the lowest values in abandoned fields and sandy plains, respectively (Table 1).

On the other hand, all the measured reproductive traits (the number of flowers, fruits, seed, and fruit dry mass) were higher in the population of the abandoned fields habitat, while the lowest values of these parameters were recorded in the mountain ranges habitat. The number of fruits, flowers, seed, and fruit dry mass in the abandoned fields habitat were 8.4-, 3.6-, 2.9-, and 2.7-fold higher than those determined for the population in mountain ranges habitat (Table 1). 

**Figure 2:**
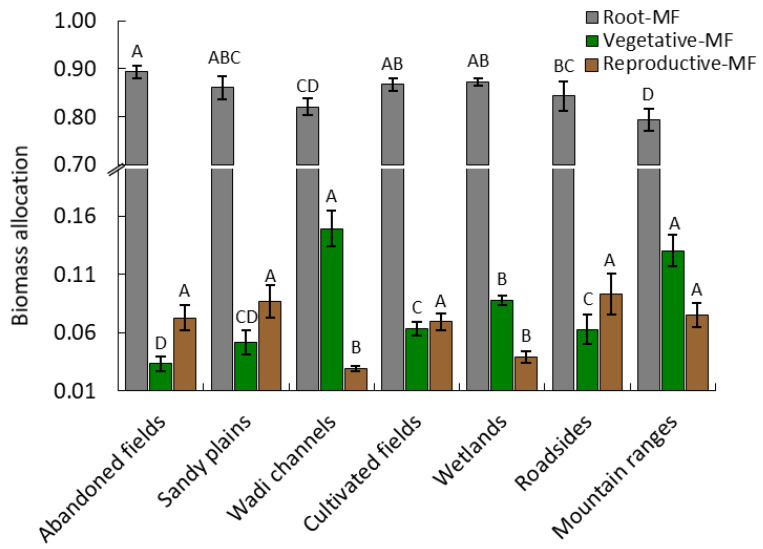
Biomass allocation of root, vegetative, and reproductive mass fraction (MF) based on the dry matter (g) of *Argemone ochroleuca* within the different habitats. Values are means (*n =* 10) and the bars represent the standard deviation. Within each parameter, values with the same letter are not significantly different at the 0.05 level using Tukey’s test.

The variation of dry weight allocation to each organ among the different habitats is shown in Figure 2. 

Allocation to roots was slightly greater in abandoned fields compared to the other habitats (*p* < 0.05). In contrast to roots, a significant difference was determined among the reproductive allocation of *A. ochroleuca*, where the population growing in the wadi channels and mountain ranges habitats had the highest allocation in the vegetative dry weight. The allocation to vegetative organs was significantly smaller in abandoned field populations. It was remarkable that allocation to vegetative organs of *A. ochroleuca* populations in the wadi channels habitat was 4.5-fold higher than the abandoned fields habitat. The allocation in the reproductive organ (fruit) was smaller in the population growing in wadi channels and wetlands habitats, while the other habitats did not show significant difference among each other at *p* < 0.05 (Figure 2).

### 3.2. Soil Variations among the Habitats of A. ochroleuca

All studied soil variables showed a significant difference among the habitats of *A. ochroleuca*, except the pH (Table 2). The soil of abandoned fields had the highest values of silt (44.20%), clay (15.30%), EC (0.80 dS/m), OM (1.18%), Na (1.36 meq/L), K (0.41 meq/L), Ca (5.50 meq/L), Mg (0.90 meq/L), SO_4_ (4.45 meq/l), total N (1015.40 mg/kg), available N (31.38 mg/kg), total P (982.47 mg/kg), and available P (8.88 mg/kg), but this habitat had the lowest content of sand (40.5%) (Table 2). 

On the other hand, the sandy plains had the highest values of sand (89.25%), but the lowest of silt (1.25%), EC (0.09 dS/m), OM (0.17%), and sulphate (0.11 meq/L). The soils of both mountain ranges and wadi channels had the lowest contents of nitrogen (18.9 mg/kg), while soils of mountain ranges, wadi channels, and roadsides were the lowest in available phosphorus.

### 3.3. Correlation between Functional Traits and Habitats 

**Figure 3:**
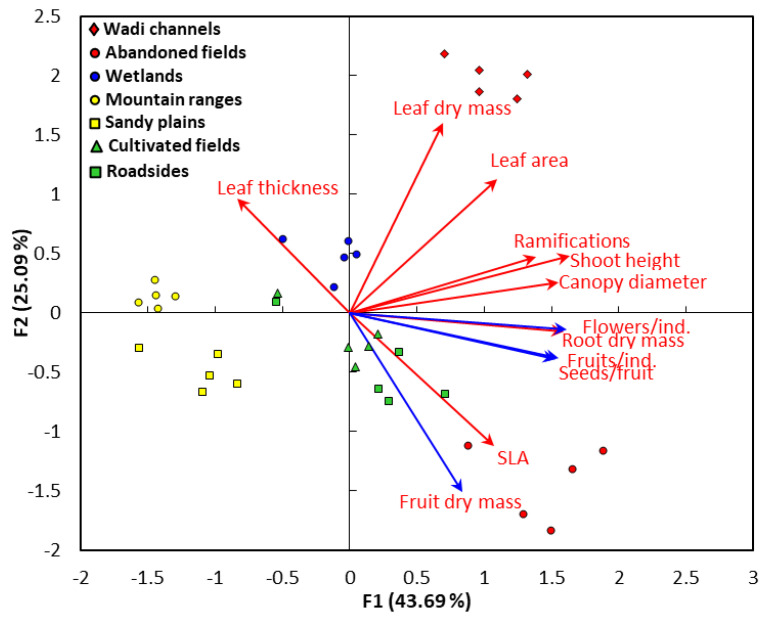
The principal component analysis (PCA) between the functional traits and different habitats of *Argemone ochroleuca*. The vegetative (red vectors) and reproductive (blue vectors) traits. SLA: specific leaf area.

The correlation analysis between the functional traits and the different habitats driven by the PCA is shown in Figure 3. It was clear that leaf area and leaf dry mass are closely correlated (Figure 3). 

Ramification, vegetative height, and canopy diameter are positively correlated to each other. On the other side, the reproductive traits: flowers/individual, fruits/individual, and seeds/individual are very closely correlated to each other. However, the leaf thickness is negatively correlated with the specific leaf area. It was evident from the data of PCA that the wadi channels habitat showed a positive significant correlation to the leaf area and leaf dry mass. On the other side, the habitat of abandoned fields showed a highly close correlation to the specific leaf area and the fruit dry mass. The wetland habitat revealed a close correlation to the leaf thickness, while the habitats of cultivated fields and roadside are overlapped and did not show a clear correlation with the functional traits. Specifically, they showed a slight correlation with fruit dry matter and the specific leaf area. Finally, the mountain ranges and sandy plains habitats are segregated together on the left part of the PCA plot and did not show a specific correlation to any trait. 

### 3.4. Correlation between Soil Variable and Habitats 

**Figure 4:**
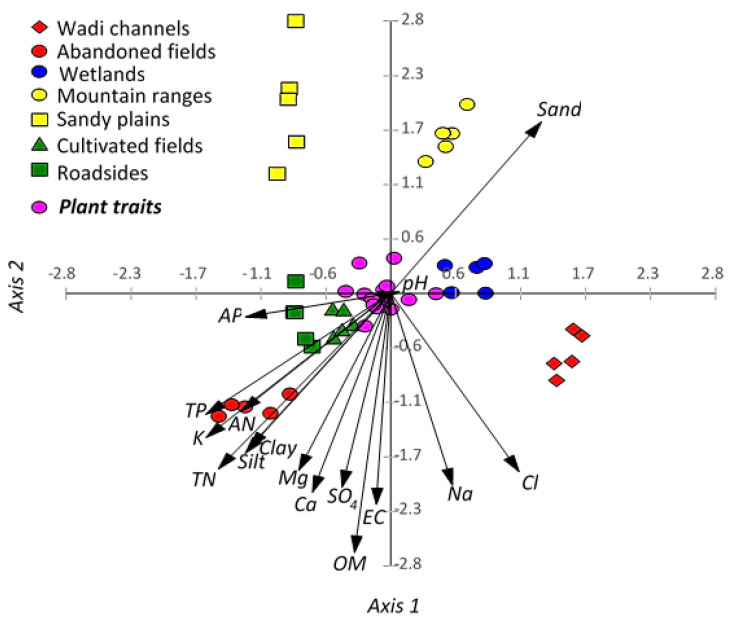
Canonical correspondence analysis (CCA) showing the correlations among the soil variables, habitats, and functional traits of *Argemone ochroleuca*. TN: total nitrogen, AN, available nitrogen, TP: total phosphorus, AP: available phosphorus, EC: electrical conductivity, and OM: organic matter.

The correlation between the habitats and the soil parameters, using CCA, revealed that most of the soil variables are correlated to each other except the sand fraction (Figure 4). The abandoned fields habitat showed a significant correlation to total N, available N, total P, silt, and clay, as well as this habitat, showed relative correlation to available P, organic matter, Ca, and Mg. However, the mountain ranges and sandy plains habitats revealed a close correlation to each other and showed a correlation to the sand fraction.

The cultivated fields, roadsides, and wetlands habitats are overlapped at the central left part of the CCA biplot and did not show a clear correlation to any parameters. Finally, the wadi channels habitat is segregated alone on the right-down part of the CCA plain and did not show a specific correlation to any variables. 

**Figure 5:**
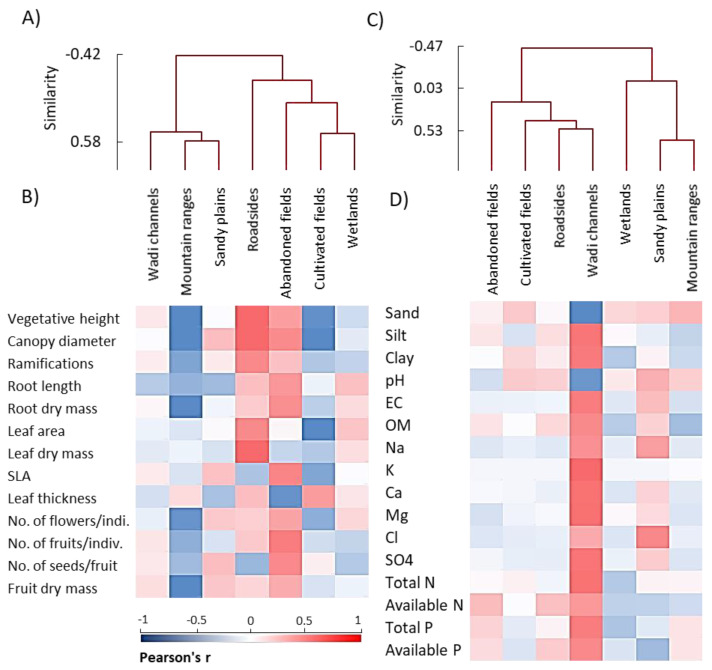
Agglomerative hierarchical clustering (AHC) and heatmaps of the studied parameters within different habitats of *Argemone ochroleuca*. (**A**) AHC and (**B**) heatmap based on the functional traits, (**C**) AHC and (**D**) heatmap based on the soil variables. EC: electrical conductivity, OM: organic matter, SLA: specific leaf area.

The AHC analysis of the functional traits of *A. ochroleuca* showed that abandoned fields, cultivated fields, and roadsides habitats were closely related, while wetlands, sandy plains, and mountain ranges habitats were similar in the vegetative and reproductive traits (Figure 5A). The wadi channels habitat was separated alone. The cluster analysis based on the soil variables of the seven studied habitats revealed that wadi channels, mountain ranges, sandy plains, and roadsides habitats were grouped together, while the cultivated fields and wetlands habitats were closely related to each other (Figure 5C). The abandoned fields habitat showed unrivaled characteristics of the soil but showed a correlation to the cultivated fields and wetland group.

Finally, the heatmap analysis showed that the populations growing in the wadi channels habitat showed positive correlations with all soil variables except sand percentage and pH, while the mountain ranges habitat showed a reversed manner (Figure 5D). On the other hand, the heatmap analysis of the functional traits revealed that *A. ochroleuca* populations growing in the abandoned fields and roadsides habitats were positively correlated with most of the functional traits (both vegetative and reproductive), while the mountain ranges and cultivated fields habitats showed negative correlations with all parameters except the leaf thickness (Figure 5B).

### 3.5. Correlation between Soil Variable and Functional Traits

Pearson’s correlation heatmap between the soil variables and the different functional traits of *A. ochroleuca* within seven habitats are shown in Figure S1. It was remarkable that most of the vegetative and reproductive traits were negatively correlated with sand, pH, and available nitrogen. The height, canopy diameter, ramification, root traits, and reproductive traits were positively correlated with EC, OM, elements, SO_4_, and total N, however, these traits were negatively correlated with sand, pH, and available P. On the other hand, the leaf traits showed a reverse manner, and interestingly, the leaf thickness showed a negative correlation to all soil variables except sand fraction. Regarding the biomass allocation, root and reproductive organs allocations revealed negative correlations with all soil variables, while the vegetative organ allocation showed positive correlations with all soil variables except for sand content and pH (Figure S1). 

## 4. Discussion

The present study revealed significant variations in all determined vegetative and reproductive traits among the habitats. These data reflect the plasticity of *A. ochroleuca*. The potential success of invasive plant species may be enhanced by their plasticity to establish and persist in different habitats through changes in their functional traits including morphological, physiological, and resource allocation traits [48]. A meta-analysis by Davidson, et al. [23] revealed that invasive species have higher phenotypic plasticity than native species, while the fitness of the non-invasive species becomes better under resource limitation. The ability to tolerate stressful conditions and to capture and utilize available resources may provide invasive species a widespread distribution and high growth rate. Additionally, several studies showed the trait plasticity of invasive species in different ecosystems [9,48,49,50,51].

On the basis of the measured functional traits, there is clear evidence that the invasion capacity of *A. ochroleuca* is largely dependent on resource availability in different habitats. The resource fluctuation hypothesis is one of the most important hypotheses of plant invasion [52], where invasive plants become facilitated by high resource availability driven by disturbance or low resource utilization by the native plant community. The nutrient-rich habitats are more vulnerable to invasion than resource-poor habitats [6,53]. However, in some cases, invasive plant species flourish in poor-nutrients habitats [54], where they employ resource conservation traits such as high resource-use efficiency.

In the present study, the wadi channels habitat had the highest values of most of the shoot parameters, i.e., vegetative height, canopy diameter, number of secondary ramifications, leaf area, leaf dry mass. On the other hand, the population of the abandoned fields habitat attained the highest values of all measured root and reproductive traits (Table 1). These data reflect fast growth and plasticity toward stressful conditions [23]. Beyond the resource availability, it is worth mentioning here that the high content of water characterizes the soil of the wadi channels habitat [55]. The water content could be an important factor for the vegetative flourishing of *A. ochroleuca* in this habitat. 

The vigorous vegetative growth of *A. ochroleuca* in wadi channels and abandoned fields compared to the drier habitats, such as sandy plains and mountain ranges, could result from enhanced soil water availability and nutrients. In arid and semi-arid regions, soil water availability, especially water pulses in streams, largely determines the growth and productivity of plant communities [55,56], particularly those of invasive herbaceous species [57]. Plant populations in habitats with low soil moisture availability and nutrients often have low growth rates and small leaves [58]. Sultan and Bazzaz [59] documented significant morphological plasticity in stem and leaf of *Polygonum persicaria* under different levels of soil moisture and nutrients.

It is worth mentioning here that the soil of the abandoned fields habitat attained high content of organic matter and nutrients (Table 2). This high resource availability could be responsible for more invasion by *A. ochroleuca*, aligned with the resource fluctuation hypothesis, which proposes that high resource availability facilitates invasion [52]. In addition, human disturbance in some habitats, such as cultivated and abandoned fields and roadsides, may increase the environmental heterogeneity that facilitates the invasibility by increasing the availability of resources, or by decreasing competition [7,60].

In contrast to the abandoned fields, the mountain ranges habitat attained the lowest measurements of almost all functional traits (Table 1). This could be attributed to low disturbance and low resources as well. Native species have more adaptability than invasive species in low resource habitats [61]. The soil of the mountain ranges habitat has very low contents of organic matter, nitrogen, and phosphorus. This agrees with the resource fluctuating hypothesis, and therefore, the native plant community in the mountain ranges habitat becomes more resistant to invasion [10]. This suggests that environmental factors, such as rainfall, topography, and soil conditions of the invaded habitats may influence the success of invasions [24,62,63]. 

Several studies indicated that many invasive plant species may achieve fast-growth through changes in below- and above-ground biomass allocation which might be associated with the successful invasion [64,65,66]. The principle of resource allocation adjustment according to habitat types as performed by *A. ochroleuca* assumes the optimization of resource partitioning in a way that maximizes fitness in variable environments [21,67] that may be timed to biotic and abiotic conditions [68]. Within each habitat, *A. ochroleuca* allocated more resources towards the vegetative and reproductive above-ground parts than to the below-ground parts which is a characteristic of many invasive species [69], and the population of abandoned fields generally had a greater resource investment in below-ground structures compared to other habitats. Such a shift in resource allocation indicates intraspecific variation and potential plasticity in plant functional traits under different environmental conditions. In old fields, Aerts and Chapin [70] argued that fast-growing invasive species are potentially increasing nutrient acquisition through increased resource allocation to roots. The adjusted resource allocation between sexual and vegetative organs in *A. ochroleuca* is likely to be important behavior in determining its colonizing ability in different habitat types. This is supported by the variations of functional traits among habitat types as an adaptive strategy to different environments [4,5].

In a comparison of the seven habitat types invaded by *A. ochroleuca*, we noticed that the population of abandoned fields had an increased root depth and biomass allocation, as well as increased SLA and thinner leaves. Such combinations of root and leaf traits may allow *A. ochroleuca* to exploit enriched soil resources in abandoned fields. These traits are typically found in fast-growing species that invade nutrient-rich sites, making them more efficient for nutrient capture [15,71]. The regulation of carbon assimilation and allocation is highly determined by SLA [58,72]. Species that have greater SLA may increase their capacity to assimilate CO_2_, and consequently call for greater amounts of resources for growth and survival [58,70].

In this context, the wadi channels habitat attained the highest vegetative allocation compared to the other habitats. This could be ascribed to the soil moisture factor, where this habitat is characterized by occasional water flowing along the wadi continuum which allows the plant to allocate more biomass toward shoots. Although the root allocation is affected more strongly by variation in soil nutrients [73], the *A. ochroleuca* showed more allocation to root in the abandoned fields habitat which attained the highest content of nitrogen. Therefore, this suggests that *A. ochroleuca* in abandoned fields was responding to combined stressful conditions; possibly salinity and anthropogenic stresses rather than to a resource limitation [74]. 

## 5. Conclusions

The success of *A. ochroleuca* as an invasive species in different habitats of arid regions seems to be related to the ability of this species to adjust its functional traits in accordance with habitat variations. The present data revealed more *A. ochroleuca* vigor in the habitat with high resource availability, which supports the resource fluctuation hypothesis. Additionally, water availability could be a factor for the vegetative flourishing of *A. ochroleuca* in wadi channel habitats. In addition, the invasion of *A. ochroleuca* seems to be more aggressive in the disturbed areas, thereby control of this invasive plant should start in the abandoned fields and the cultivated areas. Finally, the restoration of the disturbed habitats could be helpful in the control of this noxious weed.

## Figures and Tables

**Table 1:** Vegetative and reproductive traits of *Argemone ochroleuca* in different habitats in southwestern Saudi Arabia.

Traits	Habitats							*p*-Value
	Abandoned Fields	Sandy Plains	Wadi Channels	Cultivated Fields	Wetlands	Roadsides	Mountain Ranges	
**Vegetative Traits**								
Height (cm)	97.40 ± 6.58 *^A^	38.41 ± 1.88 ^CD^	115.22 ± 6.10 ^A^	72.07 ± 2.61 ^B^	57.19 ± 3.23 ^B,C^	65.04 ± 6.69 ^B^	36.80 ± 4.50 ^D^	<0.0001
Canopy diameter (cm)	75.40 ± 4.89 ^A^	39.20 ± 2.99 ^C^	81.75 ± 6.04 ^A^	53.70 ± 3.42 ^B,C^	51.51 ± 2.79 ^B,C^	66.72 ± 7.35 ^A,B^	39.11 ± 6.58 ^C^	<0.0001
Ramifications (no./individual)	7.60 ± 0.68 ^A,B^	4.29 ± 0.58 ^C,D^	9.33 ± 0.96 ^A^	6.22 ± 0.44 ^B,C^	4.60 ± 0.40 ^C,D^	6.33 ± 0.53 ^B,C^	3.40 ± 0.55 ^D^	<0.0001
Root length (cm)	26.54 ± 2.07 ^A^	17.27 ± 1.93 ^B,C^	23.22 ± 0.84 ^A,B^	14.51 ± 0.70 ^C^	23.13 ± 1.21 ^A,B^	13.38 ± 0.81 ^C^	12.80 ± 1.56 ^C^	<0.0001
Root dry mass (g)	14.43 ± 1.20 ^A^	7.37 ± 0.62 ^C,D^	11.56 ± 0.78 ^A,B^	9.50 ± 0.78 ^C^	10.78 ± 0.85 ^C^	8.91 ± 1.26 ^C^	4.56 ± 0.55 ^C^	<0.0001
Leaf area (cm^2^)	70.01 ± 5.32 ^C^	24.30 ± 2.44 ^C^	116.77 ± 5.64 ^A^	63.19 ± 7.24 ^C^	91.65 ± 3.41 ^B^	68.34 ± 5.13 ^C^	58.50 ± 11.47 ^C^	<0.0001
Leaf dry mass (mg)	529.50 ± 40.46 ^D,E^	432.55 ± 26.53 ^E^	2077.70 ± 75.14 ^A^	685.54 ± 30.75 ^C,D^	1078.19 ± 60.14 ^B^	629.18± 34.26 ^C,D,E^	743.40 ± 62.92 ^C^	<0.0001
SLA (cm^2^.g^−1^)	132.28 ± 7.98 ^A^	56.38 ± 3.58 ^E^	66.24± 4.08 ^D,E^	92.49 ± 6.05 ^B,C^	85.31 ± 4.12 ^C,D^	108.61 ± 10.99 ^B^	77.04 ± 8.28 ^C,D^	<0.0001
Leaf thickness (μm)	83.80 ± 4.34 ^E^	179.07 ± 10.21 ^A^	162.00 ± 6.62 ^A,B^	117.37 ± 5.18 ^C,D^	141.33 ± 7.68 ^B,C^	104.35 ± 5.26 ^D,E^	147.09 ± 18.75 ^B,C^	<0.0001
**Reproductive Traits**								
No. of flowers/individual	18.50 ± 1.61 ^A^	6.86 ± 0.62 ^C^	15.20 ± 1.36 ^A^	11.00 ± 0.71 ^B^	14.60 ± 1.29 ^AB^	15.64 ± 1.34 ^A^	5.20 ± 0.84 ^C^	<0.0001
No. of fruits/individual	65.36 ± 4.60 ^A^	19.41 ± 1.33 ^C^	42.86 ± 3.04 ^B^	34.31 ± 2.81 ^B^	16.87 ± 0.98 ^CD^	20.80 ± 1.07 ^C^	7.80 ± 1.03 ^D^	<0.0001
No. of seeds/fruit	495.80 ± 47.10 ^A^	320.00 ± 16.18 ^C^	427.40 ± 19.88 ^A,B^	414.40 ± 20.05 ^AB^	347.40 ± 21.50 ^BC^	452.20 ± 31.82 ^A^	173.20 ± 18.13 ^D^	<0.0001
Fruit dry mass (mg)	1151.54 ± 42.46 ^A^	728.27 ± 34.65 ^C^	410.16 ± 17.24 ^D^	748.19 ± 31.32 ^C^	475.96 ± 19.41 ^D^	931.42 ± 42.12 ^B^	426.43 ± 40.71 ^D^	<0.0001

* Values are means (*n* = 10) ± standard deviation. Within each row, means followed by the same superscript letter are not significantly different at the 0.05 level using Tukey’s test.

**Table 2:** Soil physical and chemical properties of different habitats supporting *Argemone ochroleuca* in the southwestern region of Saudi Arabia.

Soil Variable	Habitats							*p*-Value
	Abandoned Fields	Sandy Plains	Wadi Channels	Cultivated Fields	Wetlands	Roadsides	Mountain Ranges	
Sand (%)	40.50 ± 1.78 ^D^	89.25 ± 4.43 ^A^	81.80 ± 1.92 ^B^	70.53 ± 2.12 ^C^	73.06 ± 1.93 ^C^	83.50 ± 2.72 ^B^	79.52 ± 1.97 ^B^	<0.0001
Silt (%)	44.20 ± 2.34 ^A^	1.25 ± 0.03 ^E^	7.20 ± 0.84 ^D^	18.24 ± 0.92 ^B^	16.29 ± 1.25 ^B^	4.52 ± 1.13 ^D^	11.48 ± 1.15 ^C^	<0.0001
Clay (%)	15.30 ± 1.76 ^A^	9.50 ± 0.2 ^B^	11.00 ± 1.22 ^A,B^	11.23 ± 0.51 ^A,B^	10.65 ± 1.59 ^B^	11.99 ± 1.62 ^A,B^	9.00 ± 1.60 ^B^	0.003
pH	8.33 ± 0.89 ^A^	8.72 ± 0.51 ^A^	8.81 ± 0.39 ^A^	8.72 ± 0.44 ^A^	8.52 ± 0.32 ^A^	8.73 ± 0.44 ^A^	8.65 ± 0.19 ^A^	0.977
EC (dS/m)	0.80 ± 0.02 ^A^	0.09 ± 0.01 ^E^	0.51 ± 0.03 ^B^	0.17 ± 0.02 ^C^	0.16 ± 0.02 ^C,D^	0.16 ± 0.03 ^C,D^	0.12 ± 0.02 ^D,E^	<0.0001
OM (%)	1.18 ± 0.04 ^A^	0.17 ± 0.02 ^F^	0.75 ± 0.05 ^B^	0.72 ± 0.03 ^B^	0.65 ± 0.04 ^C^	0.51 ± 0.03 ^D^	0.25 ± 0.03 ^E^	<0.0001
Na (meq/L)	1.36 ± 0.03 ^A^	0.17 ± 0.03 ^C,D^	1.23 ± 0.03 ^B^	0.16 ± 0.02 ^D^	0.15 ± 0.02 ^D^	0.22 ± 0.02 ^C^	0.19 ± 0.02 ^C,D^	<0.0001
K (meq/L)	0.41 ± 0.03 ^A^	0.20 ± 0.0 ^B^	0.19 ± 0.01 ^B^	0.19 ± 0.01 ^B^	0.19 ± 0.01 ^B^	0.19 ± 0.01 ^B^	0.19 ± 0.01 ^B^	<0.0001
Ca(meq/L)	5.50 ± 0.22 ^A^	0.50 ± 0.07 ^E,F^	2.35 ± 0.09 ^B^	0.68 ± 0.09 ^D,E^	0.93 ± 0.03 ^C^	0.88 ± 0.07 ^C,D^	0.38 ± 0.05 ^F^	<0.0001
Mg (meq/L)	0.90 ± 0.06 ^A^	0.04 ± 0.01 ^D,E^	0.33 ± 0.07 ^B^	0.13 ± 0.02 ^C^	0.02 ± 0.01 ^E^	0.11 ± 0.03 ^C,D^	0.17 ± 0.04 ^C^	<0.0001
Cl (meq/L)	1.64 ± 0.08 ^B^	0.40 ± 0.02 ^C^	2.09 ± 0.02 ^A^	0.41 ± 0.03 ^C^	0.31 ± 0.04 ^D,E^	0.38 ± 0.03 ^C,D^	0.29 ± 0.03 ^E^	<0.0001
SO_4_ (meq/L)	4.45 ± 0.06 ^A^	0.11 ± 0.02 ^E^	2.04 ± 0.04 ^B^	0.31 ± 0.03 ^D^	0.52 ± 0.04 ^C^	0.34 ± 0.03 ^D^	0.39 ± 0.06 ^D^	<0.0001
Total N (mg/kg)	1015.40 ± 29.95 ^A^	508.40 ± 14.48 ^B^	515.20 ± 24.20 ^B^	447.60 ± 11.52 ^C^	489.80 ± 15.48 ^B^	521.20 ± 16.41 ^B^	297.60 ± 14.35 ^D^	<0.0001
Available N (mg/kg)	31.38 ± 1.38 ^A^	19.78 ± 3.35 ^C^	18.96 ± 3.11 ^C^	27.78 ± 5.31 ^A,B^	28.14 ± 4.73 ^A,B^	22.33 ± 3.70 ^B,C^	18.90 ± 2.56 ^C^	<0.0001
Total P(mg/kg)	982.47 ± 15.88 ^A^	845.22 ± 10.17 ^B,C^	787.61 ± 15.05 ^D^	825.54 ± 10.98 ^C^	867.54 ± 20.27 ^B^	791.36 ± 10.29 ^D^	745.43 ± 9.55 ^E^	<0.0001
Available P (mg/kg)	8.88 ± 1.21 ^A^	6.92 ± 1.14 ^A,B^	5.14 ± 1.11 ^B^	7.46 ± 1.71 ^A,B^	7.10 ± 1.63 ^A,B^	5.92 ± 1.10 ^B^	5.90 ± 1.29 ^B^	0.003 ^**^

* Values are means (*n* = 10) ± standard deviation. Within each row, means followed by the same superscript letter are not significantly different at the 0.05 level using Tukey’s test. EC: electrical conductivity, OM: organic matter.

## Supplementary Materials

The following are available online at https://www.mdpi.com/2223-7747/9/10/1268/s1, Figure S1: Pearson’s correlation heatmaps between the soil variables and the functional traits of *Argemone ochroleuca* in various studied habitats.
