# Supplementary material for: Functional Traits Plasticity of the Invasive Herb Argemone ochroleuca Sweet in Different Arid Habitats
Source: Plants (Basel). 2020 Sep 26;9(10):1268. doi: 10.3390/plants9101268 (PMC7601790; doi:10.3390/plants9101268)
Supplement: Supplementary file 1 [file plants-09-01268-s001.pdf]

plants

MDPI

Article

Functional Traits Plasticity of the Invasive Herb *Argemone ochroleuca* Sweet in Different Arid Habitats

Abdulaziz M. Assaeed [1], Saud L. Al-Rowaily [1], Magdy I. El-Bana [2], Ahmad K. Hegazy [3], Basharat A. Dar [1] and Ahmed M. Abd-ElGawad [1,4,*]

[1] Plant Production Department, College of Food & Agriculture Sciences, King Saud University, P.O. Box 2460, Riyadh 11451, Saudi Arabia; assaeed@ksu.edu.sa (A.M.A.); srowaily@ksu.edu.sa (S.L.A.-R.); baseratali@gmail.com (B.A.D.)

[2] Botany Department, Faculty of Science, Port Said University, Port Said 42511, Egypt; magdyelbana@sci.psu.edu.eg

[3] Department of Botany and Microbiology, Faculty of Science, University of Cairo, Giza 12613, Egypt; hegazy@sci.cu.edu.eg

[4] Department of Botany, Faculty of Science, Mansoura University, Mansoura 35516, Egypt

* Correspondence: aibrahim2@ksu.edu.sa; Tel.: +966-562-680-864

Received: 1 September 2020; Accepted: 24 September 2020; Published: 26 September 2020

Supplementary Materials

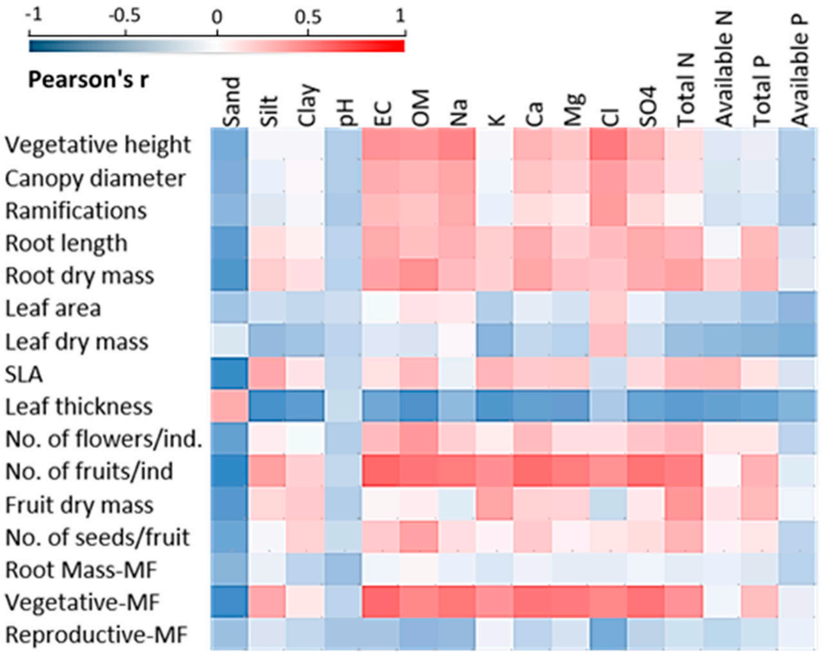

Figure S1. Pearson's correlation heatmaps between the soil variables and the functional traits of *Argemone ochroleuca* in various studied habitats. EC: electrical conductivity, OM: organic matter, SLA: specific leaf area.
